# Metabolites from Marine Sponges and Their Potential to Treat Malarial Protozoan Parasites Infection: A Systematic Review

**DOI:** 10.3390/md19030134

**Published:** 2021-02-28

**Authors:** Anna Caroline Campos Aguiar, Julia Risso Parisi, Renata Neves Granito, Lorena Ramos Freitas de Sousa, Ana Cláudia Muniz Renno, Marcos Leoni Gazarini

**Affiliations:** 1 Department of Biosciences, Federal University of São Paulo (UNIFESP), Rua Silva Jardim 136, Santos 11015-020, SP, Brazil; carolcaguiar@yahoo.com.br (A.C.C.A.); juliaparisi@outlook.com (J.R.P.); rn.granito@unifesp.br (R.N.G.); acmr_ft@yahoo.com.br (A.C.M.R.); 2 Special Academic Unit of Chemistry, Federal University of Goiás (UFG/UFCAT), Catalão Regional, Catalão 75704-020, GO, Brazil; lorennarf@ufg.br

**Keywords:** *Plasmodium*, malaria, sponge, resistance, antimalarial

## Abstract

Malaria is an infectious disease caused by protozoan parasites of the *Plasmodium* genus through the bite of female Anopheles mosquitoes, affecting 228 million people and causing 415 thousand deaths in 2018. Artemisinin-based combination therapies (ACTs) are the most recommended treatment for malaria; however, the emergence of multidrug resistance has unfortunately limited their effects and challenged the field. In this context, the ocean and its rich biodiversity have emerged as a very promising resource of bioactive compounds and secondary metabolites from different marine organisms. This systematic review of the literature focuses on the advances achieved in the search for new antimalarials from marine sponges, which are ancient organisms that developed defense mechanisms in a hostile environment. The principal inclusion criterion for analysis was articles with compounds with IC_50_ below 10 µM or 10 µg/mL against *P. falciparum* culture. The secondary metabolites identified include alkaloids, terpenoids, polyketides endoperoxides and glycosphingolipids. The structural features of active compounds selected in this review may be an interesting scaffold to inspire synthetic development of new antimalarials for selectively targeting parasite cell metabolism.

## 1. Introduction

Human malaria is an infectious disease caused by single-celled protozoan parasites of the *Plasmodium* genus (*P. *falciparum**, *P. vivax*, *P. ovale*, *P. malariae*, and *P. knowlesi*) through the bite of female *Anopheles* mosquitoes [1]. It affected 228 million people in 2018, and nearly half of the world’s population is still at risk for this disease [2]. Symptoms can range from being mild to very severe, causing chronic illness, physical disability, death and a huge health burden, especially to the most vulnerable populations. 

Antimalarials based in quinolines scaffolds (i.e., chloroquine, mefloquine, amodiaquine, and piperaquine) possess a complex mechanism of action. One well-studied mechanism involves compromising the detoxification of hemoglobin degradation with heme polymerization for hemozoin crystal formation in digestive vacuole by protonated forms of quinolones [3]. It was noted that some strains of *P. falciparum* triggered resistance to protonated drugs due to a genetic mutation in the transporter (*PfCRT*) and could lead to antimalarial drug extrusion from the organelle [3]. 

Artemisinin-based combination therapies (ACTs) are the most recommended treatment for uncomplicated *P. falciparum* malaria, while artesunate is considered the most effective antimalarial drug for severe cases [4], with several biochemical processes reported as targets in parasite cells [3,5]. Despite the safety and efficiency that have been proven for the use of these drugs, the emergence of multidrug resistance has unfortunately limited their effects and challenged the field [6]. The resistance to ACTs is already spreading from Southeast Asia, as reported in 2008 [7], giving rise to a danger alert to other high-poverty regions in the world, and the identified resistance phenotype is associated with mutation of kelch domain protein gene (k13), which is postulated to be involved in protein trafficking organelles in the parasite during intraerythrocytic cycle [8], [3].

In this context, the ocean, with its rich biodiversity, has been emerging as a very promising resource of bioactive compounds and secondary metabolites from different marine organisms (bacteria, fungi, micro-algae, mollusks and other invertebrates) with multiple pharmacological properties [9,10,11]. Among them, the phylum Porifera (sponges) is the most promising for providing raw material for the development of biotechnological products for multiple human health problems [12,13,14]. Marine sponges are very primitive sessile animals with origins dated at least from the late Proterozoic over 580 million years ago [15]. Being considered representatives of the first multicellular animals, these filter-feeding organisms evolutionarily developed morphological and chemical defense mechanisms constituted mainly by secondary metabolites, compounds with a wide range of effects such as antitumor, antiviral, anti-inflammatory and antibiotic effects, which have been investigated for the treatment of human health problems [15,16]. Additionally, some authors have demonstrated the antimalarial effects of the secondary metabolites of marine sponges and have shown that these components present inhibitory activity against the malaria parasite *Plasmodium falciparum* [6,17].

Many studies have investigated the structural diversity of marine natural products from sponges worldwide showing strong evidence of their antimalarial effects; however, there is still limited understanding of their biological effects. To explore the complete therapeutic potential of marine-sponges-derived compounds, more inputs are required, especially from the comparison of the antiplasmodial potential of all of these biocompounds. Previous reviews have contributed discussion of potential antimalarial compounds from marine sources and have helped to cover the growing number of new compounds studied every year and parasite resistance to currently used antimalarials [3,18,19]. In this context, the purpose of this study was to perform a systematic review updated of the literature to examine the multiple studies reporting the in vitro antiplasmodial activity of extracts and molecules from species of marine sponges, exploring the molecules scaffold and differential target mechanisms in cell physiology. 

## 2. Results and Discussion 

### 2.1. Study Selection and Analysis 

**Figure 1:**
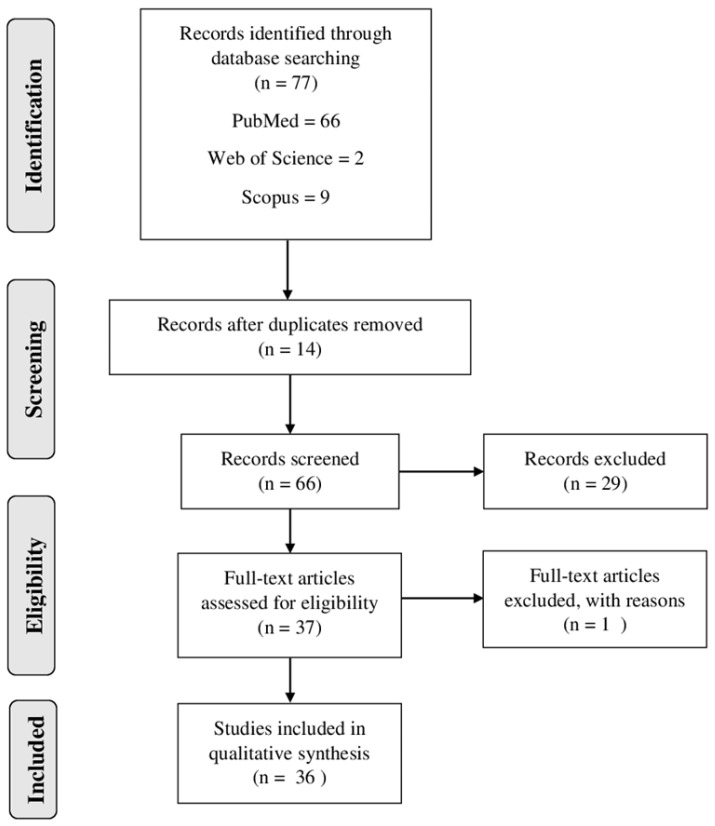
Flow diagram of literature search and selection criteria used in the present review adapted from PRISMA (Preferred Reporting Items for Systematic Reviews and Meta-Analysis).

The flow diagram (Figure 1) demonstrated the search strategy (identification, inclusion and exclusion) used in the present study. A total of 77 articles were retrieved from the databases (PubMed, Web of Science and Scopus). Then, the duplicated records were excluded (*n* = 14). Thus, 66 full-text articles were assessed for eligibility, and 30 studies were excluded for different reasons, such as the following: some studies reported only the extraction of compounds and did not report the antiplasmodial activity; others described only the mechanism of the compounds; some studies were only computational. Finally, 36 studies were included and analyzed in this systematic review (Figure 1). 

A summary of the studies is presented in Table 1. The articles analyzed were published from 1992 to 2019 in different countries. The antimalarial activity was assessed in vitro using *Plasmodium falciparum* culture [20] for quantification of cell viability over 24-96 h. For the in vitro assays, different lab strains were used (such as 3D7, W2, DD2, NF54), and a wide variety of methods were used for assessing *P. falciparum* viability ([3H] hypoxanthine, LDH, Microscopy, SYBR Green) presenting as IC_50_ values instead of the option of XC_50_. The Demospongiae sponge class was the most explored, where 30 studies evaluated their antiplasmodial activity. Among the genera in Table 1, most belong to the Demospongiae class except for *Plakortis* (*Plakortis simplexs*, *Plakortis lita*, *Plakortis halichondrioides*), which is from the Homoscleromorpha class. In addition, a great geographical variety was observed, which shows that sponges from different regions of the globe have this potential antiplasmodial activity. The inhibitory concentration for 50% of the parasites (IC_50_) varied from low micromolar to low nanomolar range, and the species *Xestospongia sp* showed the best bioactive potential, from which the compound Saringosterol was extracted, which had an IC_50_ of 0.25 nM. The individual IC_50_ for each extracted compound is reported in Table 1. The IC_50_ value units in µg/mL and ng/mL were converted to µM and nM for data comparison, and then some of compounds in Table 1, which IC_50_ was below 10 µg/mL became higher than 10 µM (see Section 3.2.2, exclusion criteria), as were compounds **10** and **11** [21], **52** [22], **2** and **3** [23], **99** [24].

To assess the study quality, we used the GRADE method [55]. The 36 studies analyzed were categorized as moderate quality (17) because (i) there were no controls in the experiments; (ii) the toxicity of the compounds was not assessed in parallel, which made it impossible to determine the selectivity of compounds; (iii) all compounds analyzed presented a high cytotoxicity, which demonstrates the unspecified use against *P. falciparum*; (iv) the methods used to measure the antiplasmodial activity were not described. A total of 19 studies were classified as being of high quality (Table S1).

**Figure 2:**
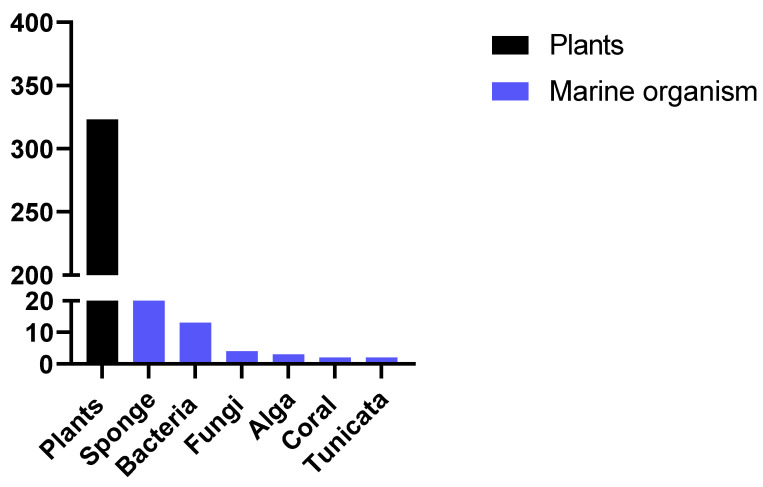
Number of published papers reporting the antimalarial activity of new compounds from marine sources or plants in the past 10 years.

After this detailed review of the articles reporting the activity of compounds from marine sponges, we made a brief survey of data in the literature to compare the number of articles published reporting the activity of marine organisms with the number of articles published reporting the activity of extracts from plants. To do so, the following combinations of keywords were used: "new antimalarials and plants" or "new antimalarials and marine" and selected the works published in the last 10 years. Figure 2 represents the number of studies reporting antiplasmodial activity of new compounds found. The search for new compounds from marine sources is still uncommon compared to the search for natural products from plants. Other recent reviews have also reported this comparison, which reinforces the importance of seeking new products from marine sources, especially considering that the diverse nature of metabolites produced by these alternative sources presents a compelling case for intensive exploration [56].

### 2.2. Classes of Compounds Found in Marine Sponge Extracts 

**Figure 3:**
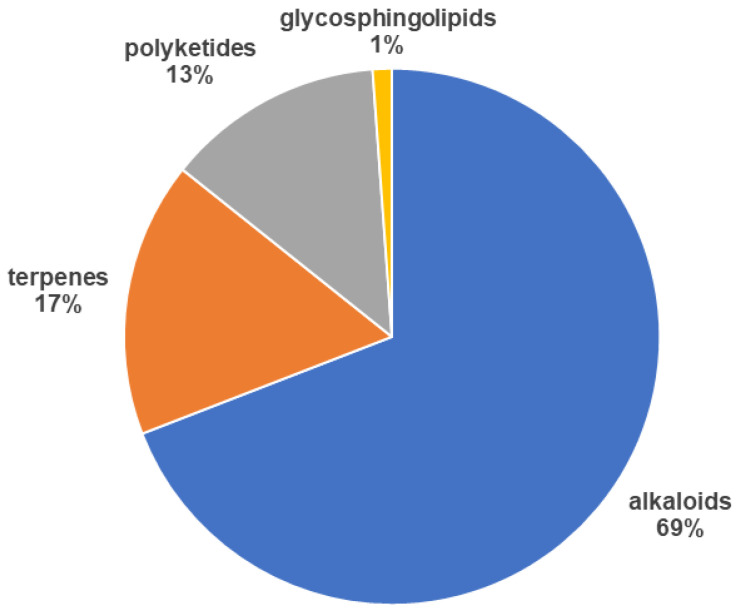
Chemical class of compounds (259) identified in the reviewed articles (37).

The compounds isolated from marine sponges presented in the articles analyzed with antiplasmodial effect belong to alkaloids, terpenes and polyketides class of secondary metabolites. Most of the compounds with potential activity against *Plasmodium* sp. are alkaloids (69% of 259), followed by terpenoids (17%) and polyketides endoperoxides (13%) (Figure 3). There are also reports of glycosphingolipids (GSL) from sponges able to inhibit the malaria parasite as well. The structures with their potency are described, and some of them present the known mechanism of action, which are discussed below.

#### 2.2.1. Alkaloids

Alkaloids from marine sponges have shown potential against infectious diseases, particularly against malaria. The natural alkaloids identified are grouped as pyrrole-imidazole [23,27], indole-imidazole [33], indole [21], manzamine [45,57], ingamine alkaloids [39], bromotyrosine [43], guanidine [28,30,32] phloeodictynes [51], pentacyclic quinones [50], pyrroloiminoquinone [38], thiazine alkaloids [34], and diterpene alkaloids [48,58].

**Figure 4:**
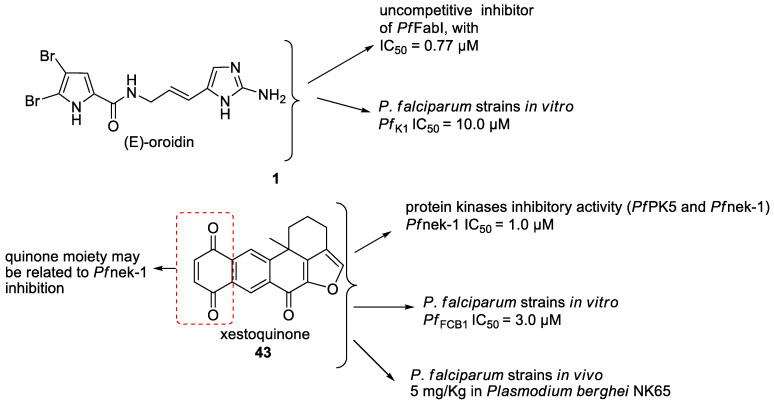
Alkaloids from marine sponges with antimalarial effect revealed moderate inhibitory activity on *P. falciparum* protein kinases (*Pf*PK5 and *Pf*nek-1) and *P. falciparum* enoyl-ACP reductase (*Pf*FabI).

Pyrrole-imidazole-related alkaloids (**1**–**3**) were verified in Demospongiae class (Porifera, horny sponges) from *Agelas oroides* (Agelasidae family) [23]. Moreover, a bromopyrrole alkaloid known as pseudoceratidine (**4**) with antiplasmodial potential (IC_50_ of 1.1 µM) was isolated from *Tedania brasiliensis* (Tedaniidae, Poecilosclerida) and *Pseudoceratina purpurea* (Pseudoceratinidae, Verongida) [27]. The (*E*)-oroidin (**1**) was a potent alkaloid against *P. falciparum* strains in vitro (IC_50_ of 10 µM), being revealed as a *Pf*FabI inhibitor (IC_50_ of 0.77 µM) with uncompetitive behavior (Figure 4) [23]. The *P. falciparum* enoyl-ACP reductase (*Pf*FabI) is an essential enzyme responsible for the catalyzes of the last step of the fatty acid pathways [59]. 

Indole alkaloids (**5**–**9**) from *Spongosorites* genus (Halichondriidae family) [33] and (*E*)-6-bromo-2’-demethyl-3’-*N*-methylaplysinopsin (**10**) and (*Z*)-6-bromo-2’-demethyl-3’-*N*-methylaplysinopsin (**11**) from *Fascaplysinopsis reticulata* (Thorectidae family) (Table 1)[21], were also shown to be inhibitors of *P. falciparum*, with nortopsentin A (**5**) as the most potent and selective compound (IC_50_ = 0.46 µM and SI 14.3). In addition, nortopsentin blocked trophozoite development, suggesting the inhibition of DNA synthesis in the early trophozoite stage [33]. 

A bioguided fractionation of Pacific marine sponge *Acanthostrongylophora ingens* (Petrosiidae family) using in vitro assay with *P. falciparum* yielded the isolation of manzamine alkaloids (**12**–**15**) (IC_50_ values between 0.010 and 0.060 µM) [45]. Manzamine A (**13**) and 8-hydroxymanzamine A (**12**) are highlighted for transcending the observed potential of antimalarial drugs in vivo on *P. berghei*-infected mice compared to chloroquine and artemisinin but with high cytotoxicity [57]. Alkaloids from *Hyrtios* Cf. erecta sponge containing β-carboline ring (**16** and **17**) but lacking polycyclic moiety were also active on *P. falciparum* in vitro [53]. Unlike manzamine A, polycyclic alkaloids without the β-carboline ring exhibited high selectivity index and maintained antimalarial effectiveness, as observed in gamine alkaloids (**18** and **19**) from *Petrosid Ng5 Sp5* [39]. 

Bromotyrosine alkaloids containing spiroisoxazoline scaffold (**20**–**26**) identified in the *Hyatella* (Spongiidae family), *Aplysinella strongylata* (Aplysinellidae family), *Pseudoceratina* (Pseudoceratinidae family) and *Verongula* genus (Aplysinidae family), have been reported as inhibitors of malaria parasite as well [40,41,43]. Among them, psammaplysin H (**20**) showed the best IC_50_ potency against 3D7 line of *P. falciparum* at 0.41 µM and the best selectivity (SI > 97) [43]. 

Guanidine alkaloids are representative antimalarial NPs [28,30,32,60], including netamines G–S from Madagascar sponge *Biemna laboutei* (Biemnidae family, Poecilosclerida) (**27**–**34**) [28,30,32] and phloeodictynes mixtures (**35**–**42**) from *Oceanapia fistulosa* [51] (Table 1). Compounds containing guanidine moiety with pentacyclic skeleton (**29**–**34**) were demonstrated to be more potent, particularly ptilomycalin F (**30**) and fromiamycalin (**34**) (IC_50_ of 0.23 and 0.24 µM, respectively) [28]. 

Pentacyclic quinone alkaloids from *Xestospongia* sp revealed moderate inhibitory activity on *P. falciparum* protein kinases (*Pf*PK5 and *Pf*nek-1) (Figure 4), enzymes involved in cell division of parasite, but xestoquinone (**43**) was able to slightly inhibit the parasite in vivo [50]. From Australian Marine sponge *Zyzzya* sp. (Acarnidae), a new compound, tsitsikammamine C (**44**), was revealed together with six known pyrroloiminoquinone alkaloids [38]. Of the seven, four were potent in vitro against resistant strains of *P. falciparum* (3D7 and Dd2, IC_50_ < 100 nM) highlighting compound **44** with high potency and lower toxicity (SI 200), which was able to act on both blood stages of parasite, ring and trophozoite [38]. Later, Davis and co-workers [34] isolated tricyclic alkaloid from *Plakortis lita* with thiazine-fused quinone, thiaplakortones A–D (**45**–**48**). Once more, alkaloids with quinone core revealed antimalarial potential in the nanomolar range (IC_50_ < 651 nM) with moderate toxicity. 

Diterpene alkaloids from *Agelas* cf. *mauritiana* (**49** and **50**) exhibited slight antimalarial potential [48], besides [58] reported a diterpene alkaloid, monamphilectine A (**51**) (*Hymeniacidon* sp.) containing a distinct β-lactam core with high potential (Table 1).

#### 2.2.2. Terpenes

Terpenes from sponges with antiplasmodial activity belong to the class of norterpene endoperoxides [17,25], sterols [25,26] meroterpenes [49], diterpenes [37,47,58], and sesquiterpenes [54,61]. Norterpene with cyclic endoperoxides scaffold is very common in *Diacarnus* genus (family Podospongiidae, order Poecilosclerida) of the marine sponges. Several norditerpene and norsesterterpene peroxide metabolites (**52**–**62**) with antimalarial potential were isolated from *Diacarnus megaspinorhabdosa* and *Diacarnus erythraeanus* species, whose peroxide moiety may be related to their activities [17,22,29]. The presence of endoperoxide in sterols from *Coscinoderma* sp., such as (24*S*)-5α,8α-epidioxy-24-methylcholesta-6-en-3β-ol (**63)** and 5α,8α-epidioxy-24-methylcholesta-6,9(11), 24(28)-trien-3β-ol (**64**), revealed activity against a resistant strain of *P. falciparum* (Dd2) as well (Table 1) [25]. Endoperoxide bridge is a pharmacophore that is well known in artemisinin drug, whose cleavage generates reactive oxygen species (ROS) inducing parasite death [62]. However, sterols from *Xestospongia* sp. (Petrosiidae family) lacking peroxide (kaimanol (**65)** and a saringosterol (**66**) were able to reduce parasite development expressively (IC_50_ values of 359 and 0.250 nM) [26].

**Figure 5:**
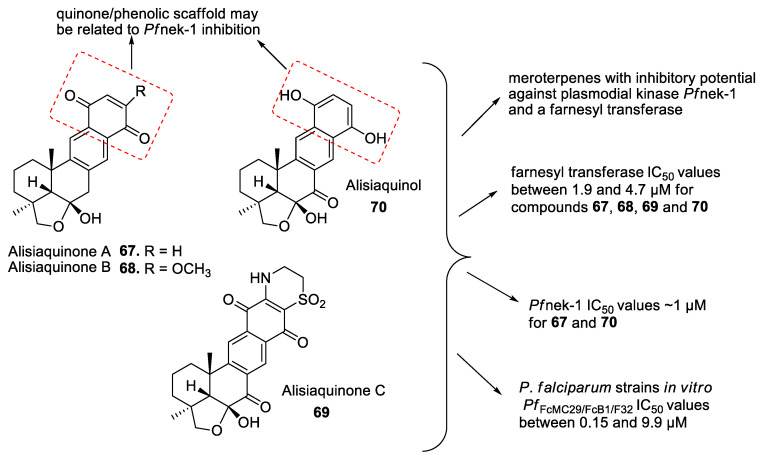
Meroterpenes from a marine sponge with antimalarial effect revealed inhibitory activity on *P. falciparum* protein kinase (*Pf*nek-1) and *P. falciparum* farnesyl transferase.

Meroterpenes (**67**–**70**) from a new Caledonian sponge with antiplasmodial effect showed inhibitory potential against plasmodial kinase *Pf*nek-1 and a farnesyl transferase (Figure 5) [49]. As we described in the section above, xestoquinone (**43**), a quinone alkaloid from *Xestospongia* sp., is also a protein kinase inhibitor (*Pf*PK5 and *Pf*nek-1), and it was suggested by Desoubzdanne and colleagues [49] that quinone/phenolic scaffold in the meroterpenes may be related to *Pf*nek-1 inhibition [50].

Diterpenes and sesquiterpenes containing isonitrile moiety with antimalarial potential have been isolated from sponges such as *Stylissa* cf. *massa* (**71**–**73**) [37], *Hymeniacidon* sp. (**74**) [58], *Cymbastela hooperi* (**75**) [47] and *Acanthella klethra* (**76**–**80**) [54] (Table 1). The isonitrile scaffold has been suggested as important for the effect of these compounds against *P. falciparum*; besides, there are sesquiterpenes lacking isonitrile moiety, as well as compounds smenotronic acid (**81**), ilimaquinone (**82**) and pelorol (**83**) from *Hyrtios erectus* with antimalarial potential (IC_50_ values ranging from 0.8 to 3.51 µM) [61].

#### 2.2.3. Polyketides

Polyketides are common secondary metabolites identified in marine sponges with vast structural diversity. Trisoxazole macrolides (**84**–**90**) are large macrocyclic polyketides from *Pachastrissa nux* (Calthropellidae family) [36,42]. The macrolides and polyketides with skeletons containing endoperoxides (six- or five-membered 1,2-dioxygenated rings), mostly found in the *Plakinastrella* and *Plakortis* genus (Plakinidae family), have been revealed to have antimalarial potential [24,31,44,52,63]. 

A series of polyketides with endoperoxides with potential against *P. falciparum* strains were isolated from *Plakortis simplex* (**91**–**97**), a Caribbean sponge (IC_50_ values ranging from 0.39 to 6.18 µM) [31,52], and from *Plakortis* sp. (**98**, **99**) and *Plakortis halichondrioides* (**100**–**105**), whose compounds **103** and **105** are endoperoxides derivatives (lactones) (IC_50_ values ranging from 0.756 to 15.1 µM) (Table 1) [24,44]. The endoperoxide moiety has been described as a pharmacophore and by computational study was suggested to be a mechanism similar to the artemisinin drug, involving radical reactions as a result of the ROS [31]. 

Derivatives of plakortin named gracilioetheres A–C from *Agelas gracilis* were isolated from a bioassay-guided approach from an active extract using *P. falciparum* assay in vitro, highlighting gracilioether B (**106**) with a IC_50_ value of 1.41 µM and moderate cytoxicity [46]. 

#### 2.2.4. Glycosphingolipids

Glycosphingolipids (GSL) are glycolipids with sugar moiety well known for the immunomodulating activity, and they have been identified in marine sponges from *Agelas* and *Axinyssa* genus [35,64]. Although there are few reports of GSL from marine sponges with antimalarial potential, Farokhi and co-workers [35] isolated a GSL with antiplasmodial activity in the low micromolar range (IC_50_ of 0.53 µM) and with low cytotoxic effect. The active mixture of GSL consists of different carbon chain lengths named axidjiferoside-A, -B and -C (**107**) from *Axinyssa djiferi* (Dictyonellidae family).

### 2.3. Mechanisms of Action of the New Compounds Found in Marine Sponge Extracts

**Figure 6:**
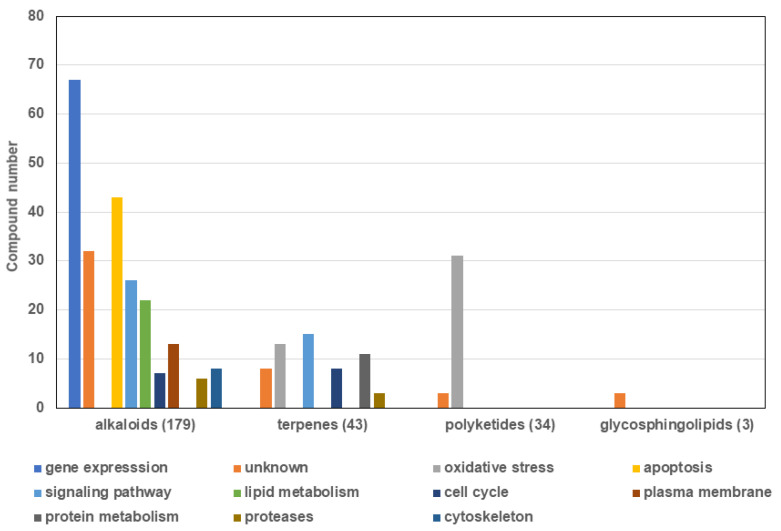
Histogram of related mechanisms of action for each chemical compound class in different cell models indicated by the literature.

We explore the mechanism of action of each class in the literature among other cell models to present a possible mechanism involved in the inhibition of *Plasmodium* development (Figure 6) because of the absence of this information in many articles described in Table 1. 

The alkaloids are the largest group of compounds mentioned in this review; however, they contain a significant number of molecules (17%) with unknown mechanisms. Some alkaloid compounds can be related with inhibition of signaling pathways, and induction of apoptosis and changes in gene expression are also indicated (14–37%) [23,65,66,67,68,69,70,71,72,73,74,75,76,77]. Alkaloids could present oxidant and antioxidant effects depending on the biosynthetic precursor. For example, bromothyrosine derivatives can induce apoptosis by the formation of reactive oxygen species or selective inhibition of histone deacetylases in eukaryotic cell lines [65]. This effect can be also observed with a marine metabolite (Psammaplin A) and analogues, resulting in disruption of the epigenetic cell control and compromising the gene expression and cell survival [73,78]. 

Quinoline analogs have been extensively studied concerning their role as the cell targets in cancer, bacteria, virus, fungi and parasites. Some of its described mechanisms are related to key cellular processes (replication, transcription, protein metabolism, etc.) because of the interaction of quinolines compounds with DNA and inhibition of topoisomerase enzymes [79,80]. Endoplasmic reticulum stress, autophagy, and cell signaling with inhibition of several enzymes (i.e., N-acetyltransferase, cyclin dependent kinase, telomerase, caspase proteases) have also been observed [77]. The impairment of cell signaling and ionic homeostasis can be observed with the antagonist effect of voltage-dependent calcium channel by guanidine derivatives alkaloids [68] and Na^+^ homeostasis by selective inhibition of *Plasmodium falciparum* P-type ATPase with indole-based natural alkaloids in a low micro-molar range [81,82]. Another important cell target is cytoskeleton filaments, which are essential for transport, cell division and organization. Some marine sponge compounds (trisoxazole-containing macrolides) can bind to F-actin subdomains by mimicking the interaction of actin-capping gelsolin family proteins, compromising the filament dynamics and leading to cell death [70,76]. The fatty acids biosynthesis is another important process for eukaryotic cells and is responsible for building membrane structures and energy metabolism. Pyrrole-imidazole alkaloids from marine sponge *Agelas oroides* present an inhibition effect at low micromolar range in *Plasmodium falciparum* enoyl-ACP reductase assay [23], which belongs to type II fatty acid pathway (FAS-II).

The second representative group is terpenes (43), which possess action related to oxidative stress and signaling pathways (30–34%) [49,50,83,84], as reported in normal and cancer cells lines, where ROS production was increased after a norterpene endoperoxide compound treatment [85]. A third group corresponds with the polyketides compounds (**34**), which have been shown to interact with Fe(II)heme, compromising the cell survival [86].

The available antimalarials (i.e., artemisinin) belong to the sesquiterpene group, and to some degree, the action mechanism of related sponge metabolites in *Plasmodium* was found to be consistent with that observed with artemisinin affecting the cell oxidative stress state and hemoglobin metabolism [3,81,87]. Hemoglobin metabolism as the principal parasite amino acid source in the host cell leads to the formation of toxic metabolites (reactive oxygen species-ROS and ferriprotoporphyrin IX). The unbalanced detoxification of these metabolites in parasite cytosol promoted by artemisinin or analogs affects many aspects of the cell physiology [81,87] as oxidative damage in different cell molecules. Some covalent protein interactions were identified with artemisinin in *P. falciparum,* indicating a broad action in cell metabolism, such as ornithine aminotransferase, pyruvate kinase, L-lactate dehydrogenase, spermidine synthase and S-adenosylmethionine synthetase [81]. In the same class of the endoperoxides, plakortin-related compounds from the sponge genus *Plakortis* bind to Fe(II) resulting in the formation of oxygen radicals and creates a cell-damaging environment for the parasite [86].

The current scenario of the development of new antimalarial drugs shows a promising molecule source from marine organisms such as sponges. However, these organisms have some weaknesses in discovering and developing antimalarial drugs: (i) the large amount of sponges’ weight needed for each compound’s identification and isolation; (ii) sponges are organisms’ symbionts with sponge-specific microbiota (unicellular eukaryotes, bacteria, fungi, virus) [88], which increases the variability from each specimen and makes it very difficult to reproduce in laboratory cultivation for identifying the source of active compounds. However, due to the ancient relationship with the hostile environment, these organisms can present a large molecule library against pathogens, which would be useful for the development of synthetic derivatives and analogs with selective inhibition of human pathogens. The cost-accessible molecular strategies available in center facilities (i.e., *high*-throughput genome sequencing and mass spectrometry, molecular docking) could surpass these limitations to the identification of compounds from complex organisms. An upscaling number of articles on marine source compounds every year presenting molecules reveals its importance with different action mechanisms in eukaryotic cell physiology, as mentioned in this review.

## 3. Methodology

### 3.1. Review Protocol

A systematic review of the literature was performed according to the SYRCLE guideline [89]. The following databases were consulted for this research: PubMed, Web of Science and Scopus. The search was carried out according to the orientations of PRISMA (Preferred Reporting Items for Systematic Reviews and Meta-Analysis). To start the review, some descriptors of the MeSH (Medical Subject Headings) were defined: “*Plasmodium falciparum*”, “*P. falciparum*”, “antimalarial” and “sponge”. In addition, two independent reviewers (J.R.P., A.C.C.A.) searched the databases, analyzing title and summary of the results, and identified them from the inclusion and exclusion criteria, and the selected studies were further reviewed during the full-text screening. 

### 3.2. Eligibility Criteria

#### 3.2.1. Inclusion Criteria

1. Studies that report the antiplasmodial activity (IC_50_) of extracts and molecules from marine sponges against any strain of *P. falciparum* in vitro;

2. Any method for determining the IC_50_ was included (SYBR Green, Hypoxanthine, Microscopy, ELISA);

#### 3.2.2. Exclusion Criteria

1. Animal experiments, clinical trials, reviews, case reports;

2. Studies that reported an IC_50_ value above 10 µM or 10 µg/mL;

3. Studies of chemical synthesis of new derivatives that were previously extracted from marine sponges;

4. Computational studies that did not report in vitro biological activity.

### 3.3. Data Extraction 

The analyzed data included the IC_50_ value, which refers to the 50% growth inhibition of the parasite in vitro after incubation with different natural products extracted from marine sponges, according to the method applied to measure the antimalarial activity with the particular *Plasmodium* lab strain used. In addition, the sponge species, class and extraction location were also included in the analysis.

### 3.4. Types of Reported Results 

Due to the heterogeneity of the primary studies, it was not possible to perform a meta-analysis. In order to compare the effect size (ES) of both techniques, we calculated the normalized average difference considering the values before and after the intervention. They were further classified as small (<0.20), moderate (about 0.50) or large (>0.80), according to Cohen criteria.

## 4. Conclusions

In conclusion, marine sponge extracts represent a large arsenal of bioactive products with antimalarial potential. Different substances, such as alkaloids, endoperoxides (terpenes and polyketides), terpenoids and glycosphingolipids, have been isolated and identified in the extracts of different sponges around the globe. The structural features of active compounds can be an interesting core for synthetic development of new antimalarials for selectively targeting parasite cell metabolism. However, studies that aim to elucidate the mechanism of action of these new compounds are still scarce in the literature.

## Figures and Tables

**Table 1:** Summary of descriptions of characteristics of included articles.

Author	Sponge Genus	Material Collection Location	Extracted Material (*P. falciparum* Strain and IC_50_ Value)
Campos et al., (2019) [21]	*Fascaplysinopsis reticulata*	Mayotte (Indian Ocean)	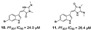
Jeong., et al. (2019) [25]	*Coscinoderma* sp.	Chuuk Island, Federated States of Micronesia	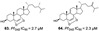
Ju et al., (2019) [25]	*Hyrtios erectus*	Chuuk Island, Federated States of Micronesia	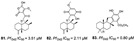
Murtihapsari. et al., (2019) [26]	*Xestospongia* sp	Kaimana, West Papua, Indonesia	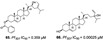
Parra et al., (2018) [27]	*Tedania Brasiliensis*	Brazil	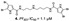
Campos et al., (2017) [28]	*Monanchora unguiculata*	Mitsio islands, Madagascar	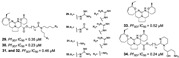
Yang et al., (2016) [29]	*Diacarnus megaspinorhabdosa*	SouthChina Sea Sponge	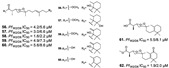
Gros et al., (2015) [30]	*Biemna laboutei*	Madagascar	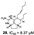
Chianese et al., (2014) [31]	*Plakortis simplexs*	South China Sea	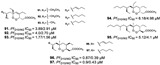
Gros et al., (2014) [32]	*Biemna laboutei*	Madagascar at Salary Ba	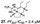
Yang et al., (2014) [17]	*Diacarnus megaspinorhabdosa*	South China Sea	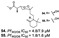
Alvarado et al., (2013) [33]	*Spongosorites* sp	Not reported	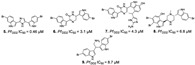
Davis et al., (2013) [34]	*Plakortis lita*	Not reported	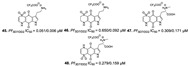
Farokhi et al., (2013) [35]	*Axinyssa djiferi*	Senegalese coasts	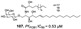
Sirirak et al., (2013) [36]	*Pachastrissa nuxs*	Thailand	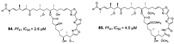
Chanthathamrongsiri et al., (2012) [37]	*Stylissa*cf. *massa*	Not reported	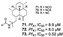
Davis et al., (2012) [38]	*Zyzzya* sp	Not reported	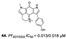
Ilias et al., (2012) [39]	*Petrosia*	Eastern Fields north of Australia	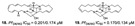
Mudianta et al., (2012) [40]	*Aplysinella strongylata*	Tulamben, Bali, Indonesia	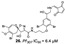
El Sayed et al., (2011) [22]	*Diacarnus erythraeanus*	Red Sea	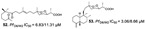
Galeano et al., (2011) [41]	*Verongula rigida*	Urabá Gulf is located in the Southwestern Caribbean	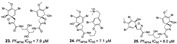
Sirirak et al., (2011) [42]	*Pachastrissa nux*	Koh-Tao, Surat-Thani ProvinceChumphon IslandsNational Park, Chumphon Province,	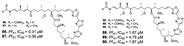
Xu et al., (2011) [43]	*Pseudoceratina* sp	Australian biota	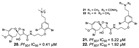
Jiménez-Romero et al., (2010) [44]	*Plakortis halichondrioides*	Puerto Rico	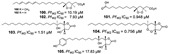
Samoylenko et al., (2009) [45]	*Acanthostrongylophora ingens*	Pacific	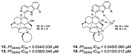
Ueoka et al., (2009) [46]	*Agelas gracilis*	southern Japan	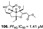
Wright et al., (2009) [47]	*Cymbastela hooperi*	Not reported	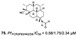
Appenzeller et al., (2008) [48]	*Agelas* cf. *mauritiana*	Solomon Islands	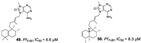
Desoubzdanne et al., (2008) [49]	New Caledonian	Norfolk Rise (New Caledonia)	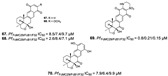
Tasdemir et al., (2007) [23]	*Agelas oroides*	Northern Aegean Sea, Turkey	- fractions: fatty acid mixtures FAME (3.4 μg/mL) and FAMF (8.7 μg/mL) 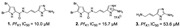
Laurent et al., (2006) [50]	*Xestospongia*	Vanuatu	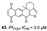
Mancini et al., (2004) [51]	*Oceanapia fistulosa*	New Caledonia Main Island	-crude mixture (0.98 μM) -*N*-methyl derivatives from the crude mixture (8 μM) 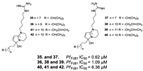
Fattorusso et al., (2002) [52]	*Plakortis simplex*	Berry Island (Bahamas)	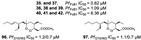
Gochfeld et al., (2001) [24]	*Plakortis* sp.	Jamaica	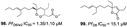
Kirsch et al., (2000) [53]	*Hyrtios* cf. *erecta*	Fiji	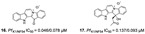
Angerhofer et al., (1992) [54]	*Acanthella klethra*	Australia	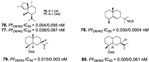

## Supplementary Materials

The following are available online at https://www.mdpi.com/1660-3397/19/3/134/s1. Table S1: GRADE analysis of included articles.
