# Supplementary material for: Metabolites from Marine Sponges and Their Potential to Treat Malarial Protozoan Parasites Infection: A Systematic Review
Source: Mar Drugs. 2021 Feb 28;19(3):134. doi: 10.3390/md19030134 (PMC7997450; doi:10.3390/md19030134)
Supplement: Supplementary file 1 [file marinedrugs-19-00134-s001.pdf]

Review

Metabolites from marine sponges and their potential to treat malarial protozoan parasites infection : a systematic review

Anna Caroline Campos Aguiar [1], Julia Parisi [1], Renata Neves Granito [1], Lorena Ramos Freitas de Sousa [2], Ana Cláudia Muniz Renno [1], Marcos Leoni Gazarini [1*]

[1] Department of Biosciences, Federal University of São Paulo (UNIFESP), Rua Silva Jardim, 136, Santos, SP, 11015020, Brazil.

[2] Special Academic Unit of Chemistry, Federal University of Goiás (UFG/UFCAT), Catalão Regional, 75704-020 Catalão, GO, Brazil

* Correspondence: marcos.gazarini@unifesp.br; Tel.: +5513 3229 0132

Received: date; Accepted: date; Published: date

Supplementary Table 1: GRADE analysis of included articles.

**GRADE Factors**								
**Author**	**Study limitation**	**Inconsistency**	**Indirectness**	**Imprecision**	**Publication bias**	**effect size**	**Dose effect**	**Overall quality**
Campos et al., (2019)[14]	✓	✓	✓	✓	X: toxicity was not assessed	✓	✓	+++
Jeong., et al (2019)[15]	✓	✓	✓	✓	X: not assessed	✓	✓	+++
Ju et al., (2019)[15]	X: did not report antimalarial control data	✓	✓	✓	✓	✓	✓	+++
Murtihapsari., et al (2019)[16]	✓	✓	✓	✓	X: toxicity was not assessed	✓	✓	+++

Parra et al., (2018)[17]	✓	✓	✓	✓	✓	✓	✓	++++
Campos et al., (2017)[18]	✓	✓	✓	✓	X: all compounds were cytotoxic with low selectivity indices	✓	✓	+++
Yang et al., (2016)[19]	✓	✓	✓	✓	X: all compounds were cytotoxic with low selectivity indices	✓	✓	+++
Gros et al., (2015)[20]	✓	✓	✓	✓	X: all compounds were cytotoxic with low selectivity indices	✓	✓	+++
Chianese et al., (2014)[21]	✓	✓	✓	✓	✓	✓	✓	++++
Gros et al., (2014)[22]	✓	✓	✓	✓	✓	✓	✓	++++
Yang et al., (2014)[13]	✓	✓	✓	✓	X: all compounds were cytotoxic with low selectivity indices	✓	✓	+++

Alvarado et al., (2013)[23]	✓	✓	✓	✓	✓	✓	✓	++++
Davis et al., (2013)[24]	✓	✓	✓	✓	✓	✓	✓	++++
Farokhi et al., (2013)[25]	✓	✓	✓	✓	✓	✓	✓	++++
Sirirak et al., (2013)[26]	✓	✓	✓	✓	X: toxicity was not assessed	✓	✓	+++
Chanthathamrongsiri et al., (2012)[27]	✓	✓	✓	✓	✓	✓	✓	++++
Davis et al., (2012)[28]	✓	✓	✓	✓	✓	✓	✓	++++
Ilias et al., (2012)[29]	✓	✓	✓	✓	✓	✓	✓	++++
Mudianta et al., (2012)[30]	✓	✓	✓	✓	X: toxicity was not assessed	✓	✓	+++
El Sayed et al., (2011)[31]	X: did ✓t report antimalarial control data; does not	✓	✓	✓	✓	✓	✓	+++

	describe the methods used for antiplasmodial evaluation and does not mention appropriate references							
Galeano et al., (2011)[32]	X: not describe the methods used for antiplasmodial evaluation and does not mention appropriate references	✓	✓	✓	✓	✓	✓	+++
Sirirak et al., (2011)[33]	✓	✓	✓	✓	✓	✓	✓	++++
Xu et al., (2011)[34]	X: not describe the methods used for antiplasmodial evaluation and does not mention appropriate references	✓	✓	✓	✓	✓	✓	+++
Jiménez-Romero et al., (2010)[35]	X: does not describe the methods used for antiplasmodial	✓	✓	✓	✓	✓	✓	+++

	evaluation and does not mention appropriate references							
Samoylenko et al., (2009)[36]	✓	✓	✓	✓	✓	✓	✓	++++
Ueoka et al., (2009)[37]	✓	✓	✓	✓	✓	✓	✓	++++
Wright et al., (2009)[38]	✓	✓	✓	✓	✓	✓	✓	++++
Appenzeller et al., (2008)[39]	✓	✓	✓	✓	✓	✓	✓	++++
Desoubzdanne et al., (2008)[40]	✓	✓	✓	✓	✓	✓	✓	++++
Tasdemir et al., (2007)[41]	✓	✓	✓	✓	✓	✓	✓	++++
Laurent et al., (2006)[42]	X: did not report antimalarial control data	✓	✓	✓	✓	✓	✓	+++
Mancini et al., (2004)[43]	✓	✓	✓	✓	✓	✓	✓	++++

Fattorusso et al., (2002)[44]	✓	✓	✓	✓	X: toxicity was not assessed	✓	✓	+++
Gochfeld et al., (2001)[45]	X:does not describe the methods used for antiplasmodial evaluation and does not mention appropriate references	✓	✓	✓	✓	✓	✓	+++
Kirsch et al., (2000)[46]	✓	✓	✓	✓	✓	✓	✓	++++
Angerhofer et al., (1992)[47]	✓	✓	✓	✓	✓	✓	✓	++++

Grade Factors: ✓: No Serious Limitations; X: Serious Limitations; For Overall Quality of Evidence: +, very low; ++, low; +++, moderate; ++++, high.
